# Comprehensive comparison and applications of different sections in investigating the microstructure and histochemistry of cereal kernels

**DOI:** 10.1186/s13007-020-0558-x

**Published:** 2020-02-01

**Authors:** Ahui Xu, Cunxu Wei

**Affiliations:** 1 Key Laboratory of Crop Genetics and Physiology of Jiangsu Province, Key Laboratory of Plant Functional Genomics of the Ministry of Education, Yangzhou University, Yangzhou, 225009 China; 2 Co-Innovation Center for Modern Production Technology of Grain Crops of Jiangsu Province/Joint International Research Laboratory of Agriculture and Agri-Product Safety of the Ministry of Education, Yangzhou University, Yangzhou, 225009 China

**Keywords:** Kernel, Sectioning method, Micromorphology, Histochemistry

## Abstract

This review summarizes the main applications of different sections and some improved sectioning methods in investigating the microstructure and histochemistry of cereal kernels. Thick sections of developing kernels prepared by free-hand and sliding microtome-aided sectioning method can be employed to elucidate tissue anatomy and histochemistry. The thin sections of mature kernels prepared by ultramicrotome-aided sectioning method can exhibit the micromorphology of starch granules when stained with iodine solution. The paraffin sections of developing kernels can exhibit the tissue anatomy of kernel, the accumulation of storage substances, and the location of protein and gene transcripts with immunohistochemistry and in situ hybridization techniques. The semithin resin sections can clearly exhibit the morphology of cells, starch granules, and protein bodies in kernel, but the sections prepared with different resins have various advantages and disadvantages for research investigating the morphology and histochemistry of cereal kernels. The improved methods of free-hand sectioning and ultramicrotome-aided sectioning of mature kernels are suitable for investigating the morphology of starch granules in a large number of samples in a short time. The modified method for preparing resin sections of whole kernels can be employed to determine the morphology and distribution of cells, starch granules, and storage protein in mature, developing, germinated, and cooked kernels in situ. This review could help researchers choose appropriate sections for investigating the microstructure and histochemistry of cereal kernels according to their study objectives.

## Background

Cereal mature kernels mainly include endosperm and embryos, and contain abundant storage starch and protein. Kernels are an important source of human staple food, animal feed, and industrial materials [1–4]. The morphology of kernels, including cells, starch granules, and protein bodies, in developing and mature kernels affects the weight, texture, and quality of kernels. Therefore, it is highly important to investigate the morphology of cereal kernels [2, 5, 6].

Separation techniques are used to prepare cells and starch granules, and their morphologies can be observed by staining and microscopy [7–9]. However, there are several disadvantages in investigating the morphology of cereal kernels using the separation technique. First, the process for the preparation of cells and starch granules is complicated. Second, cells and starch granules are easily destroyed during preparation. Third, the cells and starch granules in different regions of kernels have different morphologies and are not observed in situ using the separation technique. To avoid the problems of the separation technique, kernel sections are usually used to investigate the morphology of cereal kernels. Combined with staining and microscopic techniques, the sections can be employed to investigate the microstructure and histochemistry of cereal kernels in situ. Therefore, it is highly important to prepare sections of cereal kernels.

The thickness of kernel sections determines the resolution of the sample and its applications. According to the thickness of the section, the section can be divided into thick, thin, semithin, and ultrathin sections [10–24]. The thick section has a thickness of over 20 μm, the thin section, known as a paraffin section, has a thickness of 5 to 20 μm, the semithin section has a thickness of 0.5 to 5 μm, and the ultrathin section has a thickness of 50 to 100 nm. The ultrathin section is exclusively used in observing the ultrastructure of cells under transmission electron microscope and is not reviewed in the present paper. The thick sections prepared using the free-hand sectioning method can simply and rapidly determine the tissue anatomy and chemical composition of cereal developing kernels [10–13]. Thin sections prepared using the paraffin sectioning method are usually used to show the tissue anatomy or cell micromorphology of cereal kernels [14–16]. In addition, paraffin sections can be used for immunohistochemistry analysis of proteins and in situ hybridization of gene transcripts in kernels [17, 18]. The semithin and ultrathin sections prepared using the resin sectioning method can clearly observe the microstructure and ultrastructure of cereal kernels, respectively [4, 19–24]. Therefore, the sections prepared using different methods have different applications in investigating the microstructure and histochemistry of cereal kernels. The comprehensive comparison of different sections can help us to choose a reasonable method for investigating the microstructure and histochemistry of cereal kernels.

This review classifies sections into three categories, non-embedded section, paraffin section and resin section, according to the embedding medium and section thickness. The non-embedded sections include free-hand section, sliding microtome-aided section, and ultramicrotome-aided section. The resin sections have Historesin, Technovit 7100, Spurr, Epon 812, and LR White resin sections. The applications of these different sections are summarized and compared to investigate the microstructure and histochemistry of cells, starch granules, and protein bodies in cereal kernels. The review could provide important information for choosing a reasonable section to investigate the microstructure and histochemistry of cereal kernels according to the study objective.

## Non-embedded section

Non-embedded sections indicate that the kernels without chemical fixation and resin embedding are cut directly into thick sections using a sharp blade by hand or microtome. The non-embedded sections have free-hand sections, sliding microtome-aided sections, and ultramicrotome-aided sections.

### Free-hand section

Free-hand section means that the kernels are sliced into thick sections with a sharp metal blade by hand. The cereal developing kernels have high water content and are easily sliced into thick free-hand sections. The obtained sections are usually thick and observed under a stereomicroscope to show the tissue anatomy of kernels. In addition, the cereal mature kernels contain abundant storage starch and protein and can be sliced into thin sections. These thin sections can be observed under transmission light to show the morphology of cells, starch granules, and protein bodies. The preparation of free-hand sections from developing kernels is simple and fast, and the section of fresh sample can maintain the activities of cells or enzymes and is suitable for histochemistry analyses. In addition, the sample is not embedded and reacted easily by specific reagents in the histochemical tests.

**Fig. 1:**
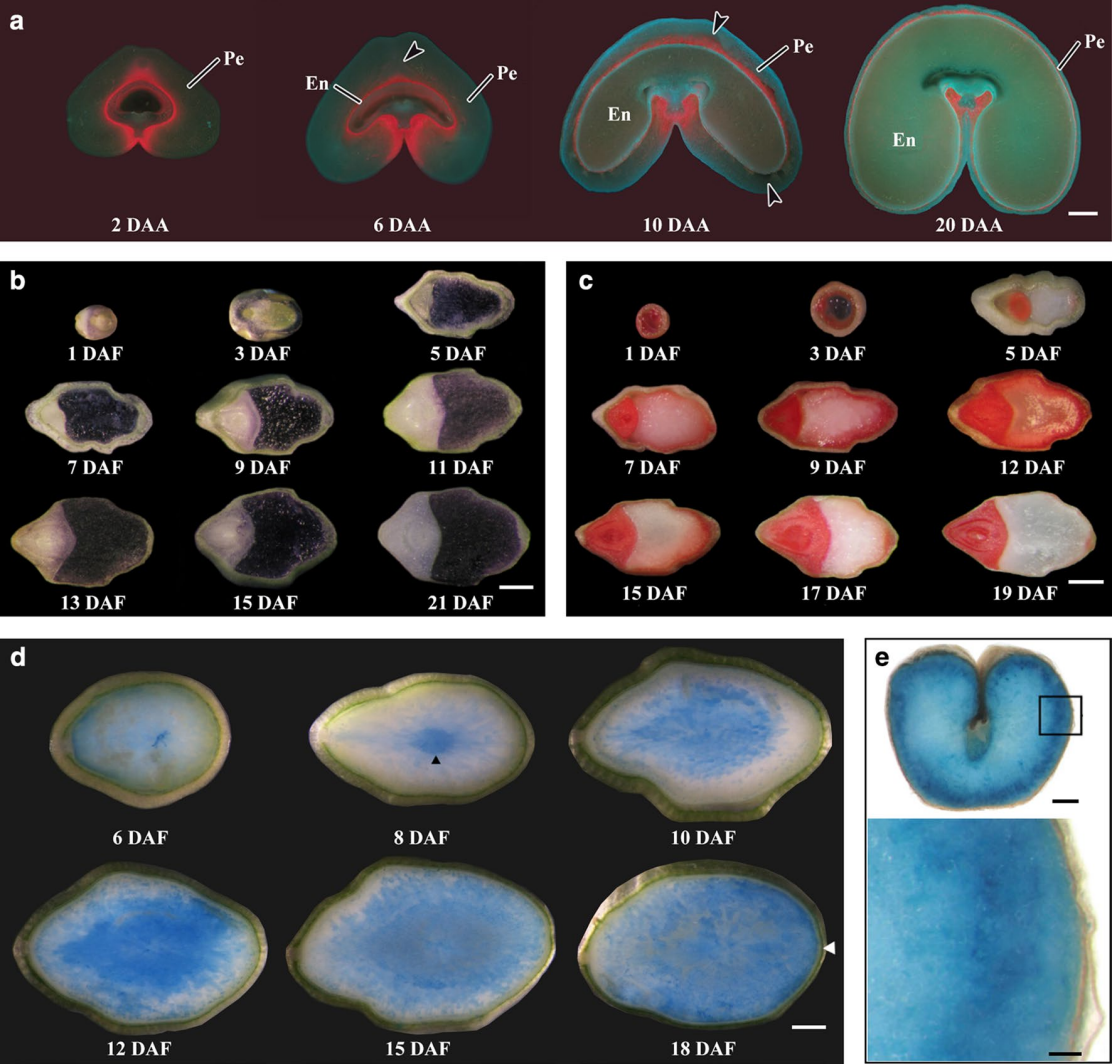
Applications of free-hand sections in cereal developing kernels. **a** Fluorescence microscope images of transverse sections of wheat developing kernels (cited from Yu et al. [13]). DAA, day after anthesis; En, endosperm; Pe, pericarp. Scale bar = 0.5 mm. **b** Vertical-section of rice developing kernels stained with iodine solution (cited from Yu et al. [11]). DAF, day after flowering. Scale bar = 1 mm. **c** Vertical-section of rice developing kernels stained with 1% triphenyltetrazolium chloride (cited from Yu et al. [11]). Scale bar = 1 mm. **d** Progressive PCD detection in rice developing endosperms stained with Evans Blue (cited from Wu et al. [25]). Scale bar = 0.5 mm. **e** Gus staining in wheat mature kernel (cited from van Herpen et al. [29]). The lower panel showing a magnification of the outermost cell layers of the upper panel. Scale bar = 0.5 mm for the whole section and 0.1 mm for region magnification

For developing kernels, the free-hand sections have some applications as below. (1) Rapid observation of the tissue anatomy of developing kernels. The dynamic changes of endosperm, embryo, and pericarp in developing kernels can be directly observed using free-hand sections of kernels under fluorescence microscope due to the autofluorescence of chloroplasts and cell wall [13] (Fig. 1a). (2) Spatiotemporal accumulation of storage materials in developing kernels. For example, Yu et al. [11, 12] prepared free-hand sections of rice and wheat developing kernels and observed the spatiotemporal accumulation of starch in the pericarp and endosperm by staining the sections with iodine solution during kernel development (Fig. 1b). (3) Viability detection of cells in developing kernels. The cells undergo proliferation, development, differentiation, and programmed death during kernel development. The viability of cells can be detected by staining the free-hand sections of developing kernels with 2,3,5-triphenyltetrazolium chloride (TTC) or Evans Blue [3, 10, 11, 25, 26]. The living cells can be stained red by TTC because the dehydrogenase in the living cells catalyzes the TTC into a red product. The embryo and aleurone cells are stained red, but the starchy endosperm cells are not stained at the late development stage of rice kernels [11] (Fig. 1c). The dead cells can be stained blue by Evans Blue, a dye that is excluded from living cells with intact plasma membrane and stains only the cytoplasm of nonviable cells [27]. For example, the pattern and progression of programmed cell death in cereal endosperm are detected by staining fresh free-hand sections of developing kernels with Evans Blue [10, 25–28] (Fig. 1d). (4) Histochemistry assay of protein in developing kernels. The histochemistry analysis of protein can be easily performed on fresh free-hand sections of developing kernels. For example, van Herpen et al. [29] used approximately 0.5-mm-thick free-hand sections of wheat kernel to detect the expression of the α-gliadin promoter by performing a histochemical assay of the GUS (beta-glucuronidase) reporter enzyme, which is driven by the α-gliadin protein promoter (Fig. 1e).

**Fig. 2:**
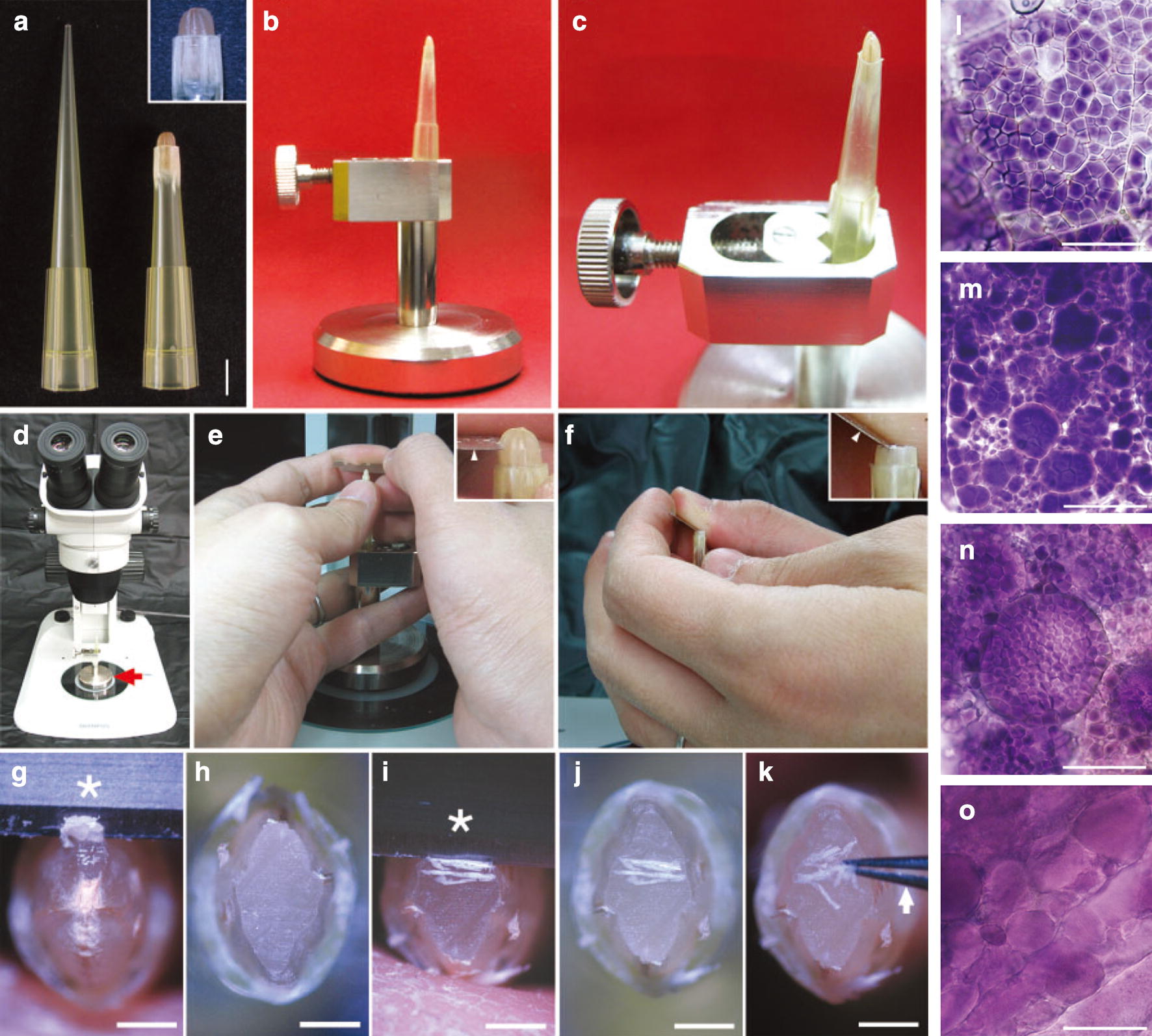
An improved method for preparing free-hand sections of cereal mature kernels and its applications in observing starch morphology (cited from Matsushima et al. [30]). **a**–**k** The preparing processes of section. **l**–**o** The morphology of starch granules in free-hand sections of mature kernels of wild type rice (**l**) and rice mutants **m**–**o** stained with iodine solution. Scale bar = 5 mm (**a**), 1 mm (**g**–**k**), and 20 μm (**l**–**o**)

The cereal mature kernels are full of starch and protein, and it is difficult to prepare their free-hand sections. Matsushima et al. [30] established a novel method for preparing thin sections of cereal mature kernels using the free-hand sectioning method (Fig. 2a–k). The sections stained with iodine solution show the shape and size of starch granules in endosperm cells. Using this method, Matsushima et al. [30] performed a screening of rice genetic populations and isolated some rice mutants with morphological defects in starch granules (Fig. 2l–o). This method is highly suitable for the examination of a large number of samples in a short time, especially for screening mutants in a genetic population, but it is difficult to control the thickness of sections and obtain fine and thin sections for mutants with chalky/floury/soft endosperm.

### Sliding microtome-aided section

Although the developing kernels can be sliced using the free-hand sectioning method, the sections are uneven and thick. To decrease the thickness of the section, a sliding microtome is used to prepare sections of cereal developing and mature kernels. For example, Wittich and Vreugdenhil [31] prepared 200-μm-thick longitudinal sections of maize developing kernels and detected sucrose synthase activity by in situ enzyme histochemistry (Additional file 1: Fig. S1A). Furukawa et al. [32] prepared 200-μm-thick sections of rice mature kernels and analyzed the distribution of storage proteins using Coomassie Brilliant Blue R250 staining and fluorescent antibody (Additional file 1: Fig. S1B).

### Ultramicrotome-aided section

**Fig. 3:**
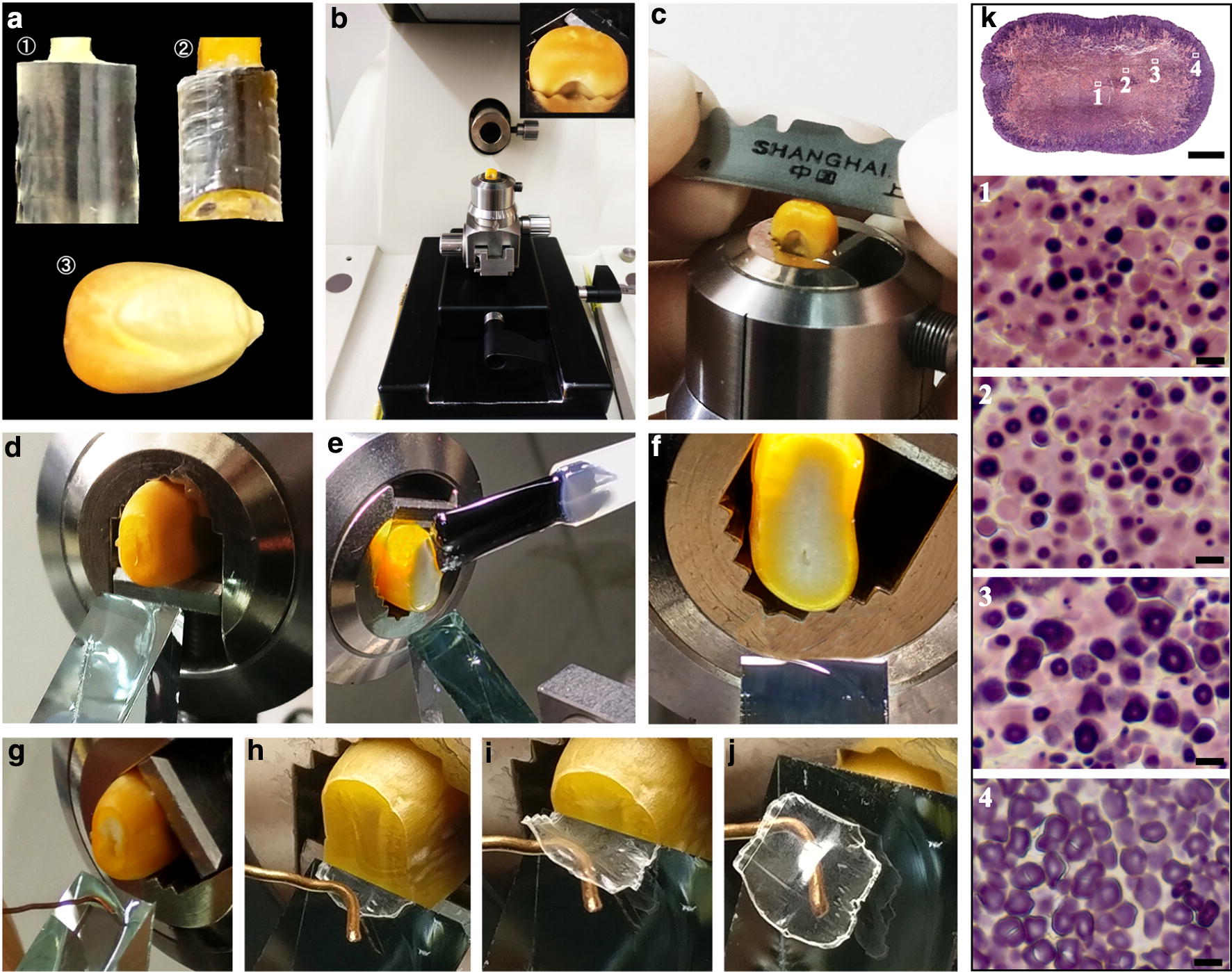
An improved method for preparing the whole section of cereal mature kernels aided with ultramicrotome and its applications in observing starch morphology (cited from Zhao et al. [35]). **a**–**j** The preparing processes of section. **e**, **f** The polished surface of chalky or floury kernel is treated with nail polish (**e**), and then be polished again after nail polish solidification (**f**). This treatment of nail polish is omitted if kernels are translucent or vitreous. **k** The morphology and distribution of heterogeneous starch granules in different regions of high-amylose maize kernels with floury endosperm. The section is stained with iodine solution, and the region 1, 2, 3 and 4 in whole section are enlarged. Scale bar = 1 mm for whole section and 10 μm for regional magnification

The even sections of developing kernels can be prepared using the sliding microtome-aided sectioning method, but the section is too thick, restricting its applications in observing the morphology of starch granules and protein bodies under light microscope. In addition, it is highly difficult to prepare thin sections of cereal mature kernels using the sliding microtome-aided sectioning method. Wellner et al. [33] prepared 2-μm semithin sections of mature maize kernels under an ultramicrotome using a glass knife. The sections stained by iodine solution can be used to observe the morphology of starch granules in endosperm cells. In addition, the sections are also used to detect the birefringence of starch granules under polarized light microscope and the structural properties of starch using Raman microscopy and infrared spectroscopy [33, 34]. However, cereal kernels with chalky/floury/soft endosperm are difficult to prepare using the ultramicrotome-aided sectioning method. Recently, Zhao et al. [35] developed a method for preparing whole sections of cereal mature kernels with translucent/vitreous/chalky/floury/soft endosperm (Fig. 3a–j). The chalky/floury/soft endosperm is treated using nail polish before sectioning with ultramicrotome. The prepared sections of mature kernels can be used to investigate the morphology and distribution of starch granules in different regions of kernels, especially for high-amylose cereal kernels enriched in heterogeneous starch granules (Fig. 3k). Therefore, the sections prepared by the ultramicrotome-aided method may be more suitable for screening mutants with heterogeneous starch granules in endosperm cells than the free-hand sections.

## Paraffin section

The free-hand and sliding microtome-aided sectioning methods can be employed to prepare the thick section of cereal developing kernels, but the sections are only suitable for analyzing the tissue anatomy of kernels under stereo microscope and cannot easily show the cell structure due to their high thickness. Although the ultramicrotome-aided sectioning method can prepare thin sections of mature kernels with hard textural structures, sections of whole kernels are difficult to obtain. In addition, continuous sections of kernels cannot be obtained using free-hand, sliding microtome-aided, and ultramicrotome-aided sectioning methods. With paraffin sections, the developing and mature kernels are embedded in paraffin, which helps to slice the kernels into evenly thin sections and continuous sections. Therefore, paraffin sections are widely used to investigate the microstructure and histochemistry of cereal kernels, although their preparation has complicated steps and is time-consuming.

**Fig. 4:**
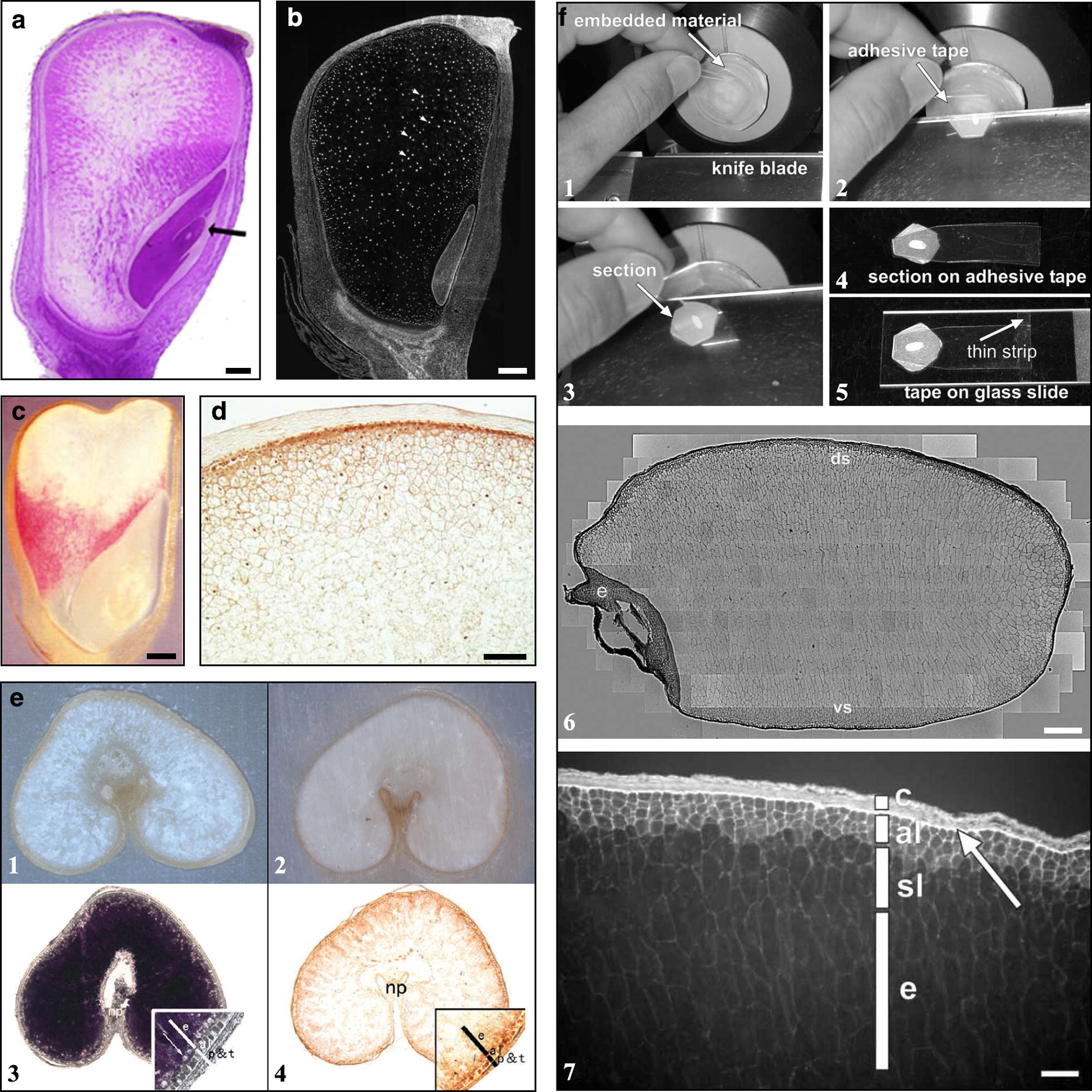
Applications of paraffin sections of cereal kernels. **a** The longitudinal section of maize developing kernels at 15 days after pollination (cited from Chen et al. [14]). The section is stained with fuchsin basic and toluidine blue. Scale bar = 0.5 mm. **b** The longitudinal section of maize developing kernels at 14 days after pollination (cited from Leiva-Neto et al. [39]). The section is stained with 4′,6-diamidino-2-phenylindole, showing the size of nuclei in different regions of kernel. Scale bar = 0.5 mm. **c** Detection of mRNA of 27-kD γ-zein in paraffin longitudinal section of maize developing kernel at 25 days after pollination (cited from Woo et al. [18]). Scale bar = 1 mm. **d** Immunohistochemical localization of δ-zein in paraffin section of maize developing kernel at 20 days after pollination (cited from Kim and Krishnan [17]). The brown color indicates the specific labeling of δ-zein on the protein bodies in subaleurone and starchy endosperm cells. Scale bar = 0.1 mm. **e** An improved method for preparing paraffin section of cereal late developing and mature kernels (cited from Zhang et al. [40]). (1) paraffin section prepared with conventional method, (2) paraffin section prepared with the improved method, (3) section of developing kernel at 35 days after pollination stained with iodine solution, showing the starch accumulation in kernel, (4) in situ localization of *bam1* transcript in the developing kernel at 35 days after pollination. **f** An improved adhesive tape method for preparing paraffin section of cereal mature kernels (cited from Ogawa et al. [41]). (1–5) the preparing processes of section, (6) reconstructed whole images of complete sections of rice mature kernel, showing cell wall arrangement and cell distribution in kernel, (7) the deparaffinized section of rice kernel at the center of the dorsal side of the kernel, showing autofluorescence. c, seed coat layer; al, aleurone layer; sl, subaleurone layer, e, starchy endosperm. Scale bar = 0.4 mm (F6) and 0.1 mm (F7)

Paraffin sections are usually used to observe the tissue anatomy or cellular micromorphology of cereal kernels, especially for early developing kernels, due to the property of continuous section. For example, Yang et al. [36] observed the process of rice embryo morphogenesis under light microscope using paraffin sections of whole embryos from 3 to 7 days after pollination. Sun et al. [37] compared the microstructure of developing endosperm and embryo between rice and its transgenic line by inhibiting the expression of the *Nuclear Factor Y gene OsNF-YB1* using 10-μm-thick paraffin sections. The tissue anatomy and cell micromorphology of maize developing kernel have also been observed using the paraffin section [14, 16] (Fig. 4a). The paraffin sections of cereal kernels can be stained with iodine solution, Coomassie Brilliant Blue and Sudan Black B to show the distribution of starch, protein and lipid, respectively [38].

Another important application of paraffin sections is the histochemistry and immunohistochemistry of cereal kernel. For example, the shape and size of nuclei in endosperm cells can be observed using paraffin section stained with 4′,6-diamidino-2-phenylindole under a fluorescence microscope [28, 39] (Fig. 4b). Woo et al. [18] analyzed the spatiotemporal expression of α-, β-, γ- and δ-zein genes in maize developing endosperm using an in situ hybridization technique on paraffin sections (Fig. 4c). Kim and Krishnan [17] detected the distribution of δ-zein in maize endosperm using immunohistochemical analysis on paraffin sections of maize developing kernel (Fig. 4d).

The cereal kernels at the late developing stage and mature stage are difficult to prepare into paraffin sections because the kernels are full of storage starch and protein and become notably hard in texture. Zhang et al. [40] developed an improved method of paraffin section to prepare the section of kernels at the late developing stage and mature stage. During the sample treatment, the chloroform replaces the xylene because the xylene causes the rapid shrinkage and hardening of samples, and the chloroform does not shrink and harden the samples. Before sectioning, the embedded kernels are softened with diethyl pyrocarbonate (DEPC) water. Using this improved method, whole sections of late developing wheat kernels are prepared. The prepared sections retain the cellular structure and antigenicity and can be used to observe the tissue anatomy and cell micromorphology, material autofluorescence, starch accumulation, protein immunohistochemistry, and gene transcript in situ localization in developing wheat kernel (Fig. 4e). For cereal mature kernels, Ogawa et al. [41] established a new technique for attaching a special pressure-sensitive adhesive tape onto the surface of paraffin-embedded tissue blocks before sectioning and then collected and placed the sections on slides such that they remained flat (Fig. 4f1–f5). Ogawa et al. [41] prepared entire longitudinal and transverse sections of mature rice kernels to observe the cell distribution and cell wall arrangement in the endosperm according to the autofluorescence of the cell wall (Figs. 4f6 and f7). In addition, the cell wall changes of cooked rice kernels are also studied using the adhesive tape method [42].

## Resin section

Although the paraffin sections can observe the tissue anatomy and histochemistry of cereal kernels, the sections are too thick for the fine structure of cells and the morphology of starch granules and protein bodies in kernels to be observed. Resin sections indicate that the samples are embedded in resin and then sliced into 1–5-μm semithin sections using a microtome. According to the embedding medium, the resin sections usually are Historesin, Technovit 7100, Spurr, Epon 812, and LR White resin sections. These resin sections have different characteristics in observing the microstructure and histochemistry of cereal kernels.

### Historesin section

Historesin is a hydroxyethyl methylacrylate and is used as an embedding medium in the form of a kit. The Historesin sections are prepared on a rotary microtome with a steel knife. The thicknesses of these sections are usually from 2 to 5 μm, which is suitable for observing the cell microstructure under light microscope [1, 43].

**Fig. 5:**
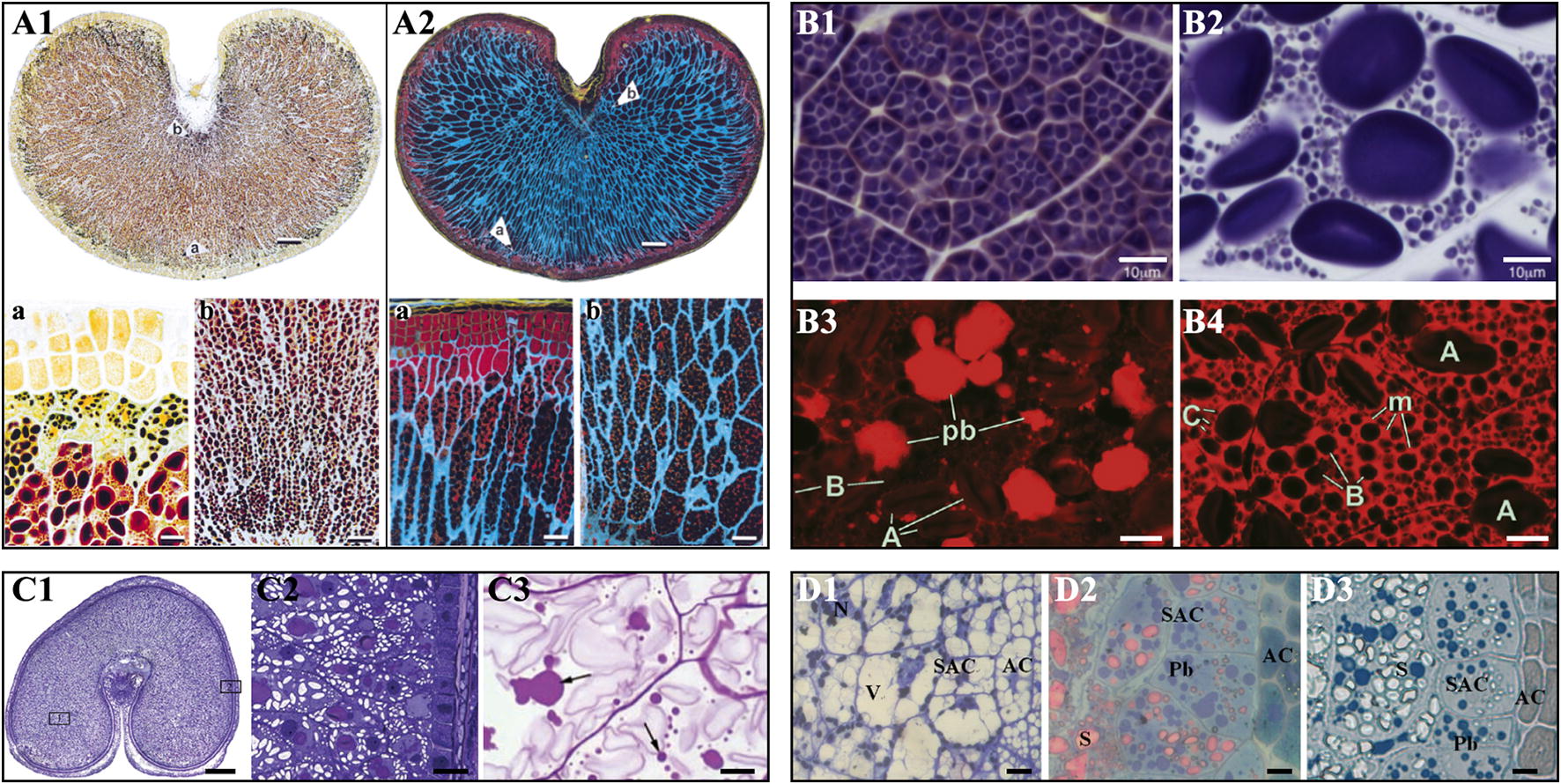
Applications of resin sections of cereal kernels. **A** Historesin sections of barley mature kernels (cited from Andersson et al. [19]). **A1** The section is stained with 0.1% light green and 50% lugol’s water solution under bright field microscope, staining protein yellow, amylose dark blue, amylopectin brown, and cell walls white (unstained). **A**2 The section is stained with 0.1% acid fuchsin and 0.01% calcofluor white M2R under fluorescence microscope, staining aleurone protein red, endosperm protein orange, cell walls blue and starch black (unstained). Scale bar = 250 μm **A1**, **A**2, 50 μm **A1**a, **A1**b, **A2**b, and 20 μm **A2**a. **B** Technovit 7100 resin sections of cereal kernels. **B1**, **B2** The sections of mature rice kernel **B1** and barley kernel **B2** are stained with iodine solution, showing the morphology of starch granules in endosperm (cited from Matsushima [45]). **B3**, **B4** The sections of wheat developing kernels at 10 days after anthesis **B**3 and 24 days after anthesis **B4** are stained with acid fuchsin, showing accumulation and distribution of storage protein (cited from Hurkman and Wood [21]). Scale bar = 20 μm **B3**, **B4**, and 10 μm **B1**, **B2**. **C** Spurr resin sections of cereal kernels. **C1**, **C2** The sections of wheat developing kernels at 22 days after anthesis are stained with toluidine blue (cited from Tosi et al. [22]). **C3** The sections of wheat developing kernels at 18 days after anthesis are stained with 0.5% methyl violet (cited from Chen et al. [49]). Scale bar = 500 μm **C1**, 50 μm **C2**, and 10 μm **C3**. **D** Epon 812 resin sections of barley developing kernels (cited from Wei et al. [50]). **D1** The sections of kernel at 8 days after heading are stained with toluidine blue; **D2** The sections of kernels at 20 days after heading are stained with periodic acid-Schiff’s reagent and azure-methylene blue; **D3** The sections of kernels at 20 days after heading are stained with Coomassie Brilliant Blue. Scale bar = 10 μm

Historesin is highly permeable such that it is suitable to prepare the whole sections of mature kernels. In addition, the sections have good dyeing ability. The Historesin sections of the whole kernel of mature barley and wheat are stained with light green and iodine solution to show protein (yellow-green) and starch granules (blue-violet) under a light microscope or acid fuchsin and calcofluor white to show protein (red) and cell walls (blue) under a fluorescence microscope [19] (Fig. 5A). The morphology and distribution of endosperm cells, starch granules, and protein bodies show significant differences between different barley and wheat varieties [1, 19]. In addition, the Historesin sections can be used for immunohistochemistry experiments to observe different types of cell wall polysaccharides. For example, Dornez et al. [43] detected the distribution of arabinoxylans and *β*-glucan using immunolabeling with monoclonal antibodies.

### Technovit 7100 resin section

Technovit 7100 is a glycol methacrylate resin, and consists of a new and less toxic initiator-accelerator chemical. The Technovit 7100 embedding solution can only be formulated before use because its polymerization process begins as soon as the three components are combined and completed within 2 h at room temperature. The sample needs to be cut into 1-mm^3^ sections due to the short polymerization time of the Technovit 7100 embedding kit [44].

Although the Technovit 7100 resin section is not suitable for ultrastructure observation under an electron microscope due to instability and brittleness under an electron beam, it can be used for observation of the cell microstructure under a light microscope [44, 45]. The morphology of starch granules in endosperm cells can be clearly observed in 1-μm-thick Technovit 7100 resin sections stained with iodine solution [30, 45-47] (Fig. 5B1, B2). To investigate the effects of high temperature on protein deposition in developing wheat kernels, Hurkman and Wood (2011) [21] prepared Technovit 7100 sections stained with acid fuchsin to investigate the morphology and distribution of protein bodies in endosperm cells under a fluorescence microscope (Fig. 5B3, B4). The results show that the high temperature during the kernel filling stage can alter the morphology and number of protein bodies in the endosperm, affecting the yield and quality of cereal crops.

### Spurr resin section

Spurr resin (also known as ERL 4206) is an epoxy resin. This resin has low molecular weight, low viscosity, small volume shrinkage after polymerization, and the ability to withstand electron bombardment for a long time in a vacuum. In addition, this resin penetrates into plant cells easily and can preserve cellular structure notably well. Therefore, semithin sections of Spurr resin have been widely used in morphological observations of cereal kernels.

The micromorphology of developing endosperm is widely observed in Spurr resin sections stained with toluidine blue [10, 12, 22, 48] (Fig. 5C1, C2). The microscopic structures of mature kernels are also observed in Spurr sections stained with toluidine blue [22]. The accumulation and distribution of protein bodies in developing endosperm is observed in Spurr resin sections stained with methyl violet. The results show that the number of protein bodies changes in the endosperm under drought stress [49] (Fig. 5C3).

### Epon 812 resin section

Epon 812 resin is another epoxy resin, and has similar properties to those of Spurr resin. However, compared with Spurr resin, the Epon 812 resin has high viscosity, restricting its application in morphology observation of cereal kernel. The Epon 812 semithin sections of cereal kernels can be stained with toluidine blue, periodic acid-Schiff’s reagent, azuremethylene blue, and Coomassie Brilliant Blue to exhibit the morphology of cells, starch granules, and protein bodies in developing endosperm [50] (Fig. 5D). The Epon 812 resin is usually used to prepare ultrathin sections to observe the ultrastructure of cells under a transmission electron microscope.

### LR White resin section

LR White resin is an acrylic resin and has low viscosity and strong permeability, leading to its wide applications in micromorphology observation of cereal kernels, especially for large sample tissues and mature kernels [4, 23].

The LR White resin sections have good stain ability and can be stained by many chemical dyes for microstructure observation under light and fluorescent microscope [4]. LR White resin sections of cereal endosperm can be stained with periodic acid-Schiff’s reagent, Coomassie Brilliant Blue, iodine solution, amino black 10B, and toluidine blue to exhibit the micromorphology of cells, starch granules, and protein bodies under light microscope [4, 23-25, 51] (Additional file 1: Fig. S2A–C). For starch observation, the iodine solution is the best choice [20, 52-54] (Additional file 1: Fig. S3). The LR White resin sections can be stained with fluorescent brightener 28 and acid fuchsin to exhibit the cell wall and protein bodies under a fluorescence microscope, respectively [4, 23] (Additional file 1: Fig. S4). LR White resin sections can maintain protein antigenicity and have applications in immunocytochemistry [55]. For example, Palmer et al. [56] observed and compared the location and dynamics of cell wall polysaccharides in both developing wheat and rice kernels using immunofluorescence detection on LR White resin sections (Additional file 1: Fig. S2D), and Tosi et al. [22] observed different types of storage proteins in wheat endosperm using immunolocalization on LR White resin sections (Additional file 1: Fig. S2E).

**Fig. 6:**
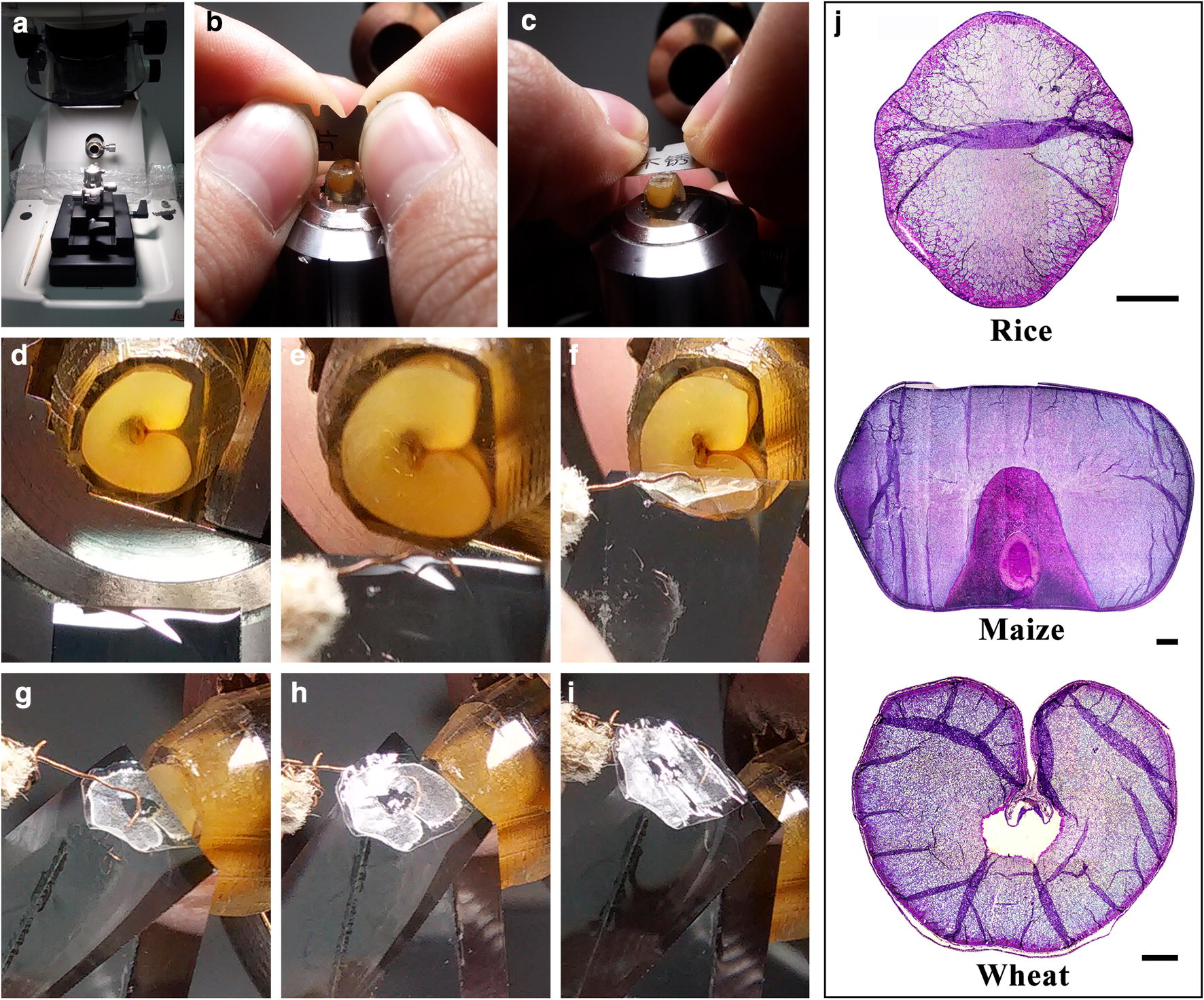
An improved method for preparing the whole section of cereal mature kernels embedded in LR White resin (cited from Zhao et al. [4]). **a**–**i** The preparing processes of section. **j** The prepared whole transverse sections of rice, maize and wheat mature kernels stained with 1% safranin O and 0.5% methyl violet. Scale bar = 500 μm

The sections of cereal mature and whole kernels are highly important for the in situ investigations of endosperm. Zhao et al. [4] established a whole-section method of cereal mature kernels embedded in LR White resin (Fig. 6). Using the whole-section method, the mature and developing kernels of rice variety Te-qing (TQ) and its transgenic resistant starch (TRS) line with inhibition of starch branching enzyme are sliced into 2-μm semithin LR White resin section. The sections stained with iodine solution clearly indicate that the polygonal, aggregate, elongated, and hollow starch granules in TRS are regionally distributed from the interior to the exterior of endosperm [20] (Additional file 1: Fig. S3), and these heterogeneous starch granules form before 10 days after flowering [57]. Xu et al. [23] prepared transverse and longitudinal LR White resin sections of maize whole kernels with different vitreousness and stained them with fluorescent brightener 28, iodine solution, and acid fuchsin to exhibit the morphology of cells, starch granules, and protein bodies, respectively, in different regions of mature endosperm. The in situ observation shows that the morphology of endosperm cells and starch granules is not related to the texture of endosperm, while the distribution of storage protein is positively correlated with the vitreousness of endosperm (Additional file 1: Fig. S4), which provides a reference for the quality breeding and application of maize kernels with different vitreousness. Zhao et al. [24] investigated the spatiotemporal accumulation and characteristics of starch in developing maize kernels using the LR White resin section of whole kernel stained with iodine solution. The developing endosperm accumulates starch from the interior to the exterior of kernel in the transverse direction and from the top to the bottom of kernel in the longitudinal direction. Some typical compound starch granules are detected in the interior of the endosperm (Additional file 1: Fig. S5). The scutellum of the embryo accumulates starch from 10 days after pollination. The pericarp starch forms and then degrades from the top to the bottom with pericarp development [24]. The fine morphology changes of starch granules can also be clearly observed in situ in cooked and germinated rice kernels using LR White resin section of whole kernel [58, 59] (Additional file 1: Figs. S6, S7). The endosperm starch in normal rice is gelatinized from the exterior to the interior of the kernel and undergoes volume swelling, shape change, granule adhesion, and amylose release during cooking (Additional file 1: Fig. S6). However, the various starch granules in the endosperm of high-amylose rice have different shape variations and gelatinization resistance during cooking [58]. The starch in the endosperm of normal rice is degraded from the exterior to the interior of the kernel and from the proximal to the distal region of the embryo during seedling development. However, the starch in the endosperm of high-amylose rice shows different degradation patterns (Additional file 1: Fig. S7).

## Conclusions and application perspectives

Sections are commonly used to observe the morphological structure and changes of tissues and cells under normal growing environments, adversity stress, and pathological conditions. This review summarizes the applications of non-embedded section, paraffin section, and resin sections in investigating the morphology and histochemistry of cereal developing and mature kernels. The non-embedded section includes free-hand, sliding microtome-aided, and ultramicrotome-aided sections.

In this study, several important and commonly used sections with considerable application prospects are again emphasized. (1) Free-hand section. The kernel development directly determines the yield and quality of cereal crops. The distribution of storage materials and the activity of cells or certain enzymes in developing kernels can be quickly detected at the tissue anatomy level using free-hand sections. (2) Improved ultramicrotome-aided section. Cereal seed mutants are important genetic resources in crop breeding programmes but usually have chalky/floury/soft endosperm. For kernels with chalky/floury/soft endosperm, the improved ultramicrotome-aided method with nail polish treatment can prepare the complete section of whole kernel to observe the morphology and distribution of starch granules in different regions of the endosperm in situ and provides a simple and rapid process for screening cereal seed mutants from a large genetic mutant population in a short time. (3) Paraffin section. The paraffin section method can prepare continuous sections of samples and is widely used to investigate the process of tissue morphogenesis. In addition, the paraffin section can perform immunohistochemistry and in situ hybridization experiments to determine the location of the targeted protein and gene transcripts at the tissue level. (4) Resin semithin section. The resin semithin section can clearly exhibit the morphology of cells, starch granules, and protein bodies in kernels due to its higher resolution. The LR White resin section not only has strong stainability but also retains the antigenicity of protein for immunohistochemistry experiments. (5) Improved resin section. The improved method for preparing the resin section of whole kernels can observe the morphology and distribution of cells, starch granules and storage protein in kernels in situ. For example, using the improved resin section, the spatiotemporal accumulation and morphology characteristics of starch granules are observed during maize kernel development [24], the morphology and distribution of heterogeneous starch granules are detected in different regions of high-amylose rice mature kernels [20], and the morphology changes of starch granules are investigated in situ in cooked and germinated rice kernels [58, 59]. In conclusion, the chosen appropriate section is highly important for investigating the micromorphology and histochemistry of cereal kernels according to the study objective.

## Supplementary information

**Additional file 1: Fig. S1.** Applications of sliding microtome-aided sections of cereal kernels. **Fig. S2.** Applications of LR White resin sections of cereal kernels. **Fig. S3.** Spatial distribution of heterogeneous starch granules in mature kernels of high-amylose rice with inhibition of starch branching enzyme I and IIb. **Fig. S4.** Morphological characteristics of cells, starch granules, and protein bodies in different regions of maize mature kernel. **Fig. S5.** Accumulation and morphology of starch granules in different regions of maize developing kernel. **Fig. S6.** Changes in morphology of starch granules in different regions of rice kernels during cooking process. **Fig. S7.** In situ degradation of starch granules in endosperm of rice at different days after imbibition.

## Data Availability

Not applicable.
