# Supplementary material for: Comprehensive comparison and applications of different sections in investigating the microstructure and histochemistry of cereal kernels
Source: Plant Methods. 2020 Feb 1;16:8. doi: 10.1186/s13007-020-0558-x (PMC6995210; doi:10.1186/s13007-020-0558-x)
Supplement: Supplementary file 1 — Additional file 1: Fig. S1. Applications of sliding microtome-aided sections of cereal kernels. Fig. S2. Applications of LR White resin sections of cereal kernels. Fig. S3. Spatial distribution of heterogeneous starch granules in mature kernels of high-amylose rice with inhibition of starch branching enzyme I and IIb. Fig. S4. Morphological characteristics of cells, starch granules, and protein bodies in different regions of maize mature kernel. Fig. S5. Accumulation and morphology of starch granules in different regions of maize developing kernel. Fig. S6. Changes in morphology of starch granules in different regions of rice kernels during cooking process. Fig. S7. In situ degradation of starch granules in endosperm of rice at different days after imbibition. [file 13007_2020_558_MOESM1_ESM.pdf]

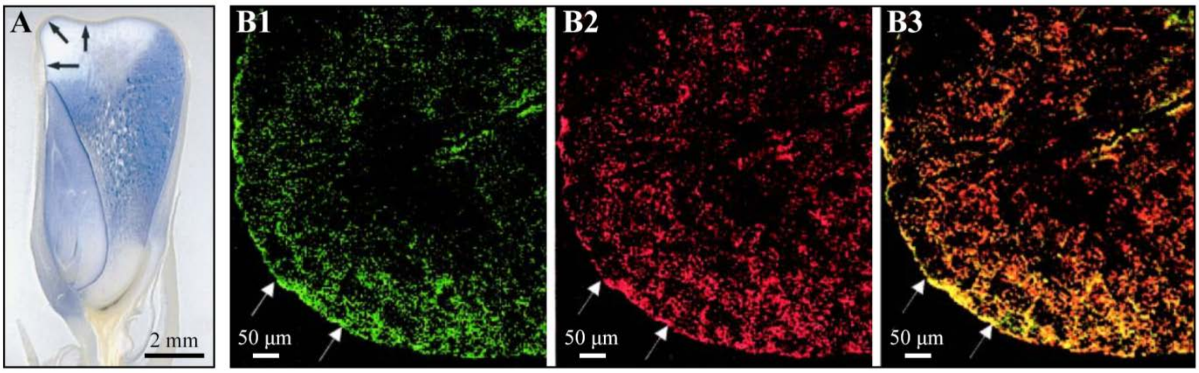

Additional file 1: Figure S1. Applications of sliding microtome-aided sections of cereal kernels. (A) Detection of sucrose synthase activity in maize developing kernel at 19 days after pollination (cited from Wittich and Vreugdenhil [31]). (B) Distribution of protein bodies in rice mature kernel (cited from Furukawa et al. [32]). The protein body I (prolamin) (B1) and protein body II (glutelin) (B2) in endosperm are exhibited through different fluorescence labeling, and their co-localization distribution is shown in (B3).

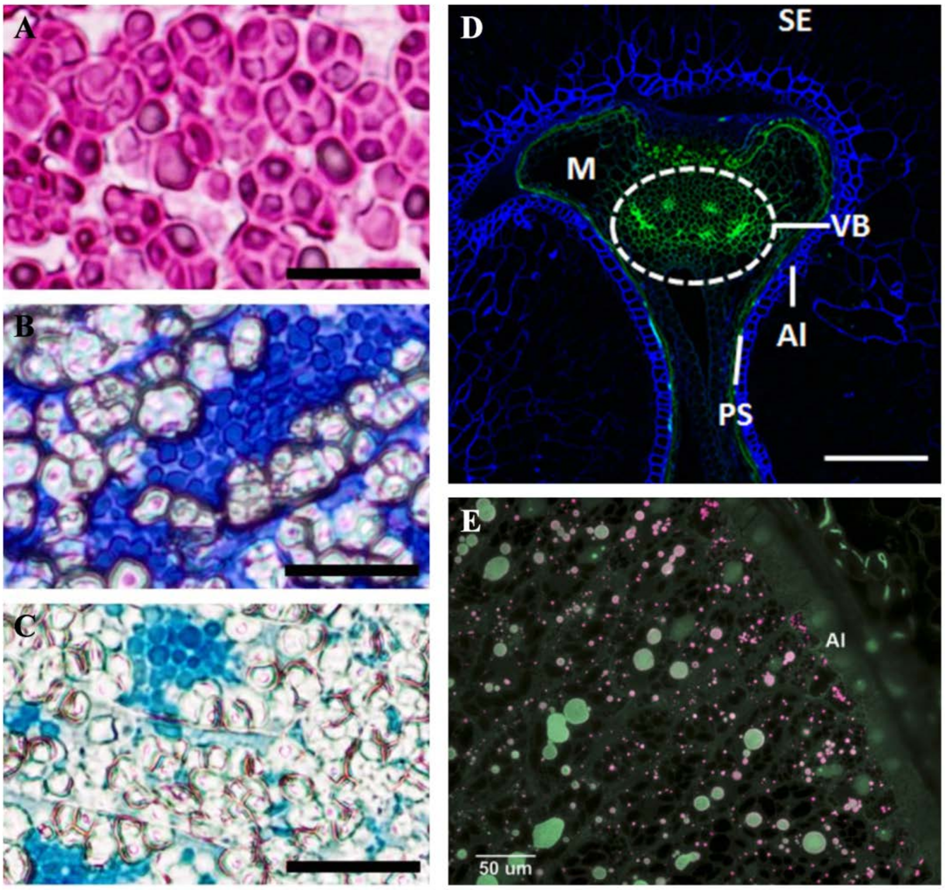

Additional file 1: Figure S2. Applications of LR White resin sections of cereal kernels. (A-C) Sections of rice mature kernel stained with periodic acid-Schiff's reagent (A), coomassie brilliant blue R250 (B), and amino black 10B (C), exhibiting starch granules and protein bodies in endosperm (cited from Zhao et al. [4]). Scale bar = 30 µm. (D) Immunofluorescence detection of glucuronoxylan in medial transverse section of wheat developing kernel at 28 days after anthesis (cited from Palmer et al. [56]). The section is counterstained with calcofluor white solution. Al, aleurone; M, maternal pericarp; PS, pigment strand; SE, starchy endosperm; VB, vascular bundle. Scale bar = 200 µm. (E) Immunofluorescence double labelling of section of wheat developing kernel at 22 days after anthesis (cited from Tosi et al. [22]). The magenta and green show the locations of the gliadin and glutenin in endosperm, respectively. Scale bar = 50 µm.

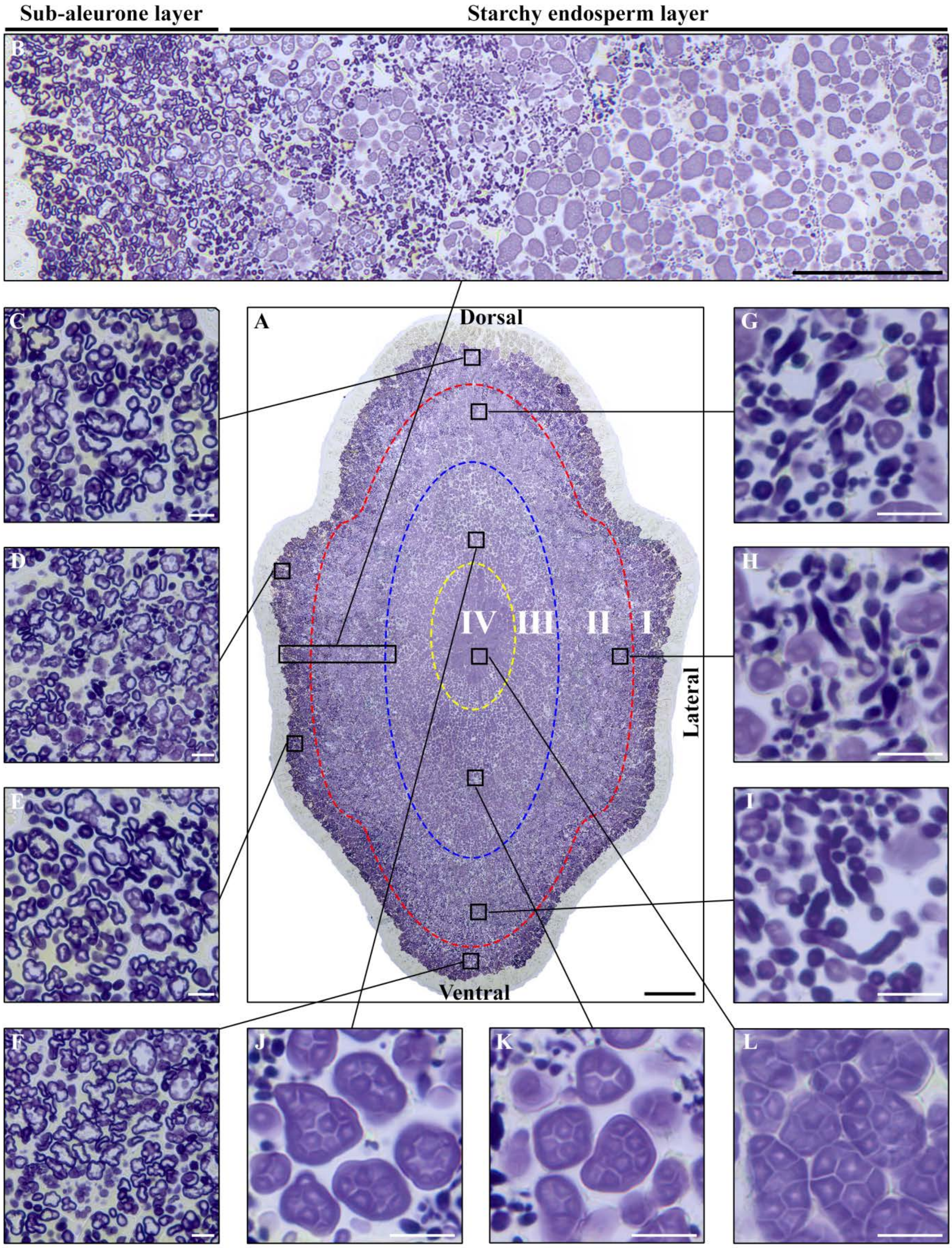

Additional file 1: Figure S3. Spatial distribution of heterogeneous starch granules in mature kernels of high-amylose rice with inhibition of starch branching enzyme I and IIb (cited from Cai et al. [20]). The LR White resin section is prepared using the improved method of whole section of mature kernel, and stained with iodine solution. The heterogeneous starch granules incude hollow (C-F), elongated (G-I), aggregate (J, K), and polygonal granules (L). Scale bar = 200 μm (A), 100 μm (B), and 10 μm (C-L).

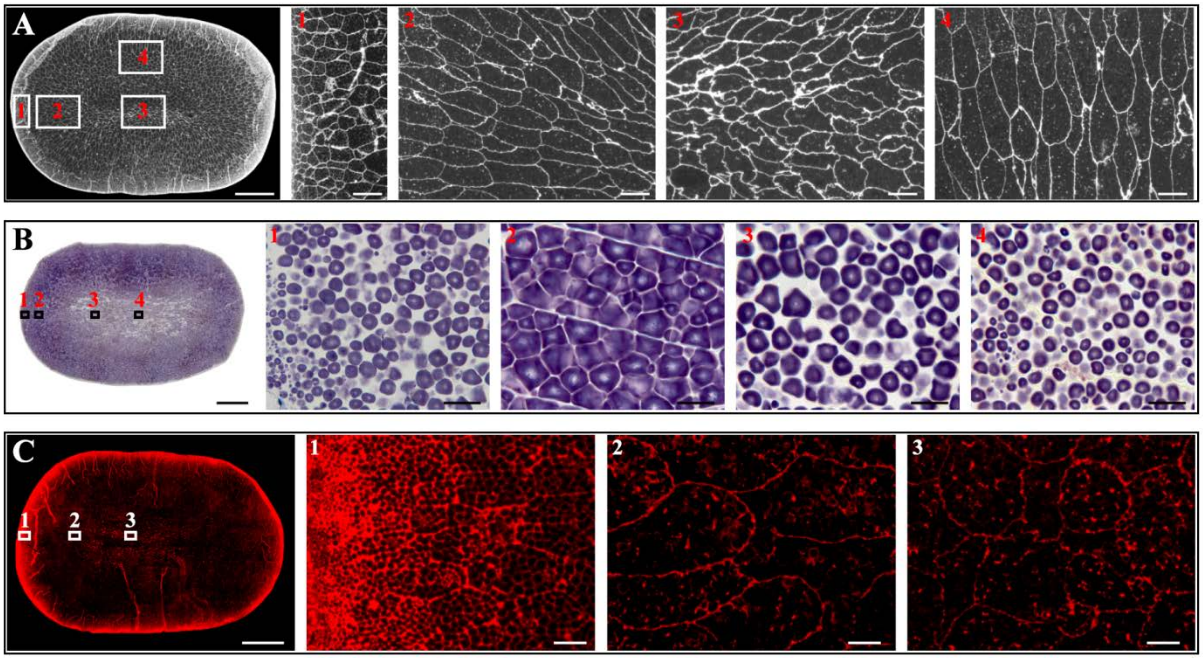

Additional file 1: Figure S4. Morphological characteristics of cells, starch granules, and protein bodies in different regions of maize mature kernel (cited from Xu et al. [23]). The LR White resin is prepared using the improved method of whole section of mature kernel. (A) Fluorescent micrographs of transverse sections of kernels stained with fluorescent brightener 28, exhibiting cell wall and showing the shape and size of cells. (B) Sections stained with iodine solution, showing starch granules. (C) Section stained with acid fuchsin, showing storage protein distribution. The magnified regions of the image is labelled in the whole section. Scale bar = 1 mm for whole section, 10 μm for region magnification (A), 20 μm for region magnification (B), and 40 μm for region magnification (C).

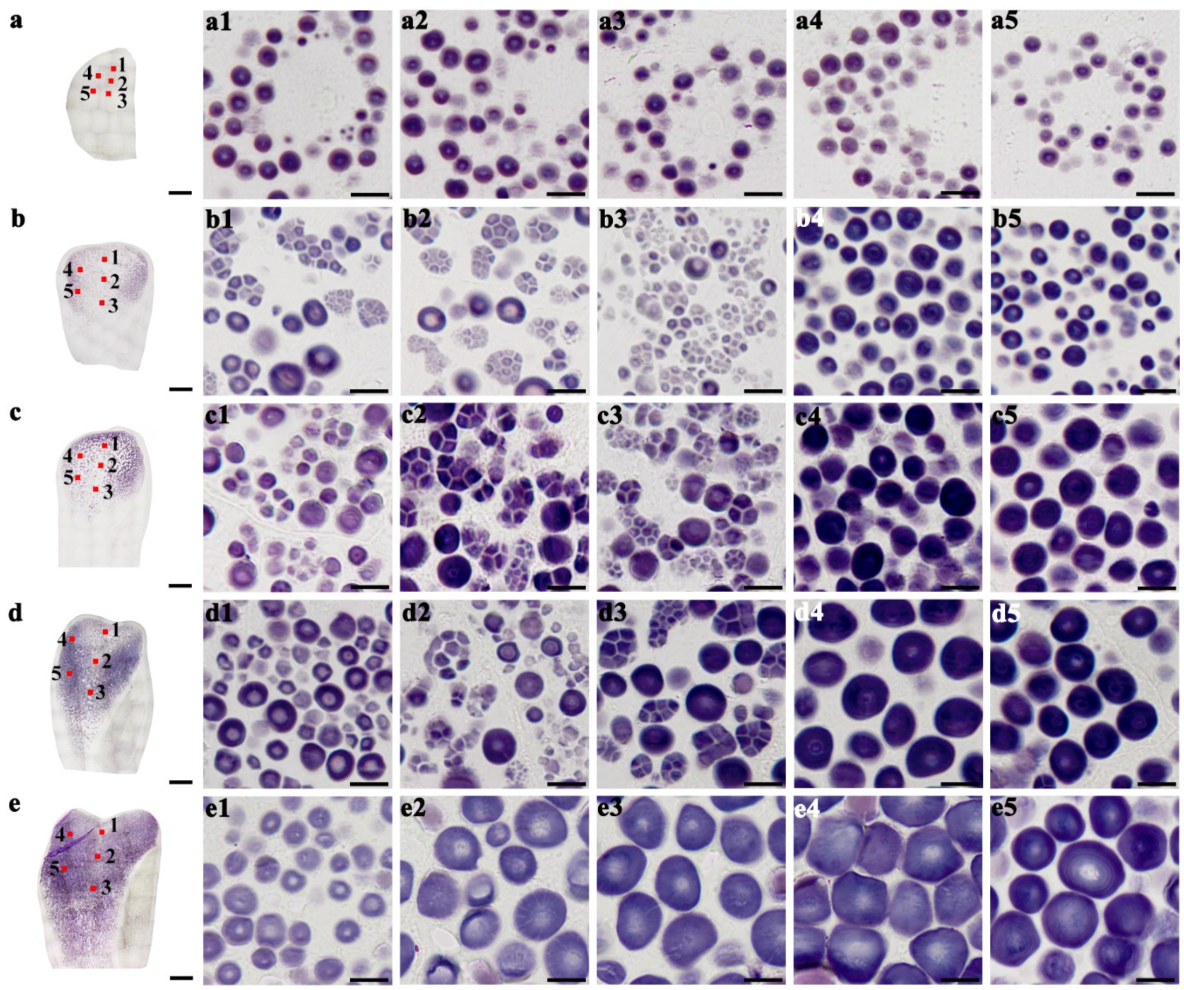

Additional file 1: Figure S5. Accumulation and morphology of starch granules in different regions of maize developing kernel (cited from Zhao et al. [24]). The LR White resin is prepared using the improved method of whole section of mature kernel, and stained with iodine solution. The magnified regions of the image is labelled in the whole section. Scale bar = 1 mm (a-e) and 10 μm (a1-e5).

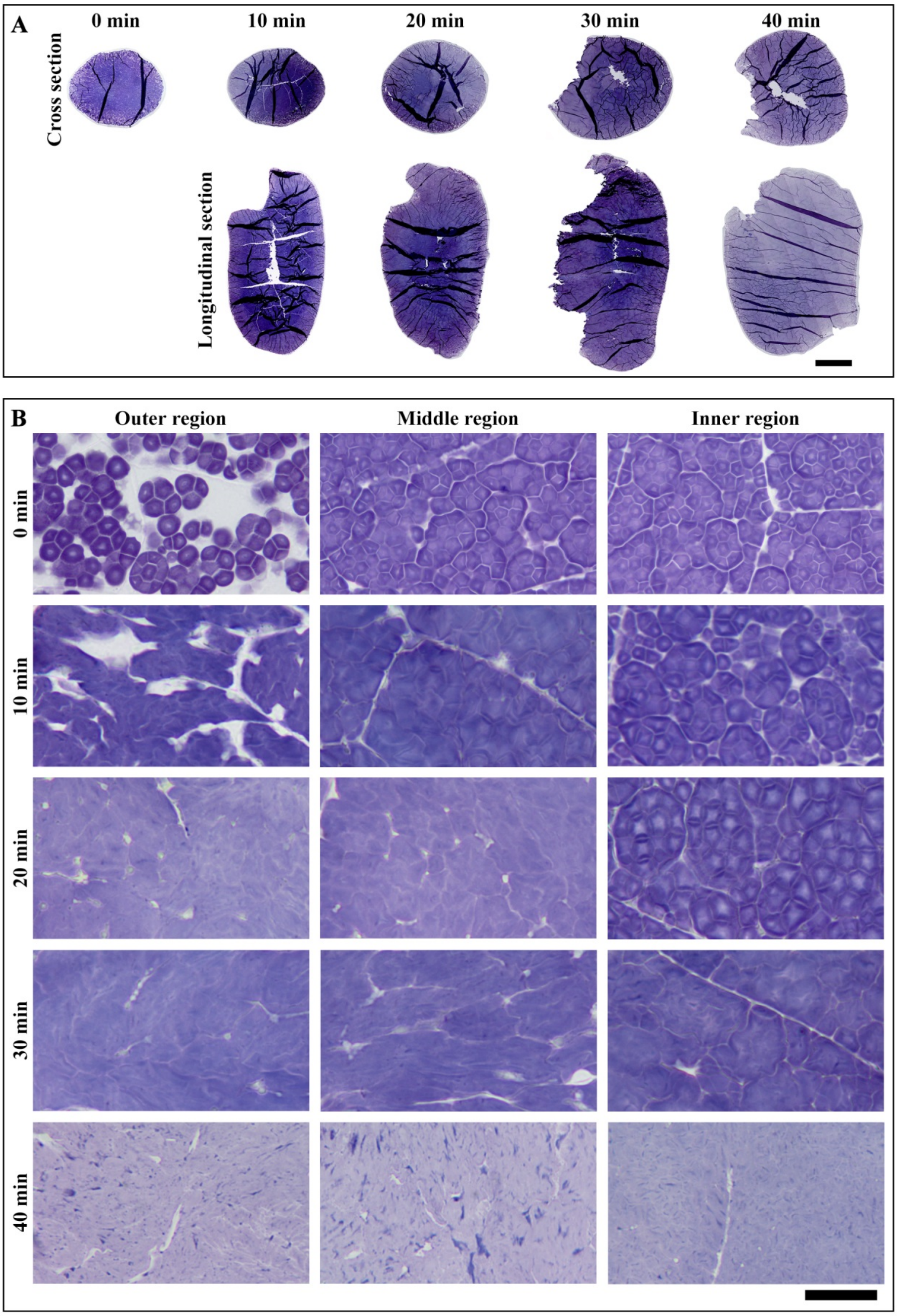

Additional file 1: Figure S6. Changes in morphology of starch granules in different regions of rice kernels during cooking process (cited from Pan et al. [58]). The LR White resin section is prepared using the improved method of whole section of mature kernel, and stained with iodine solution. (A) The cross and longitudinal sections of whole kernels for cooking of 0, 10, 20, 30, and 40 min. (B) The morphology of starch granules in different regions of cooking kernels. Scale bar = 1 mm (A) and 20 μm (B).

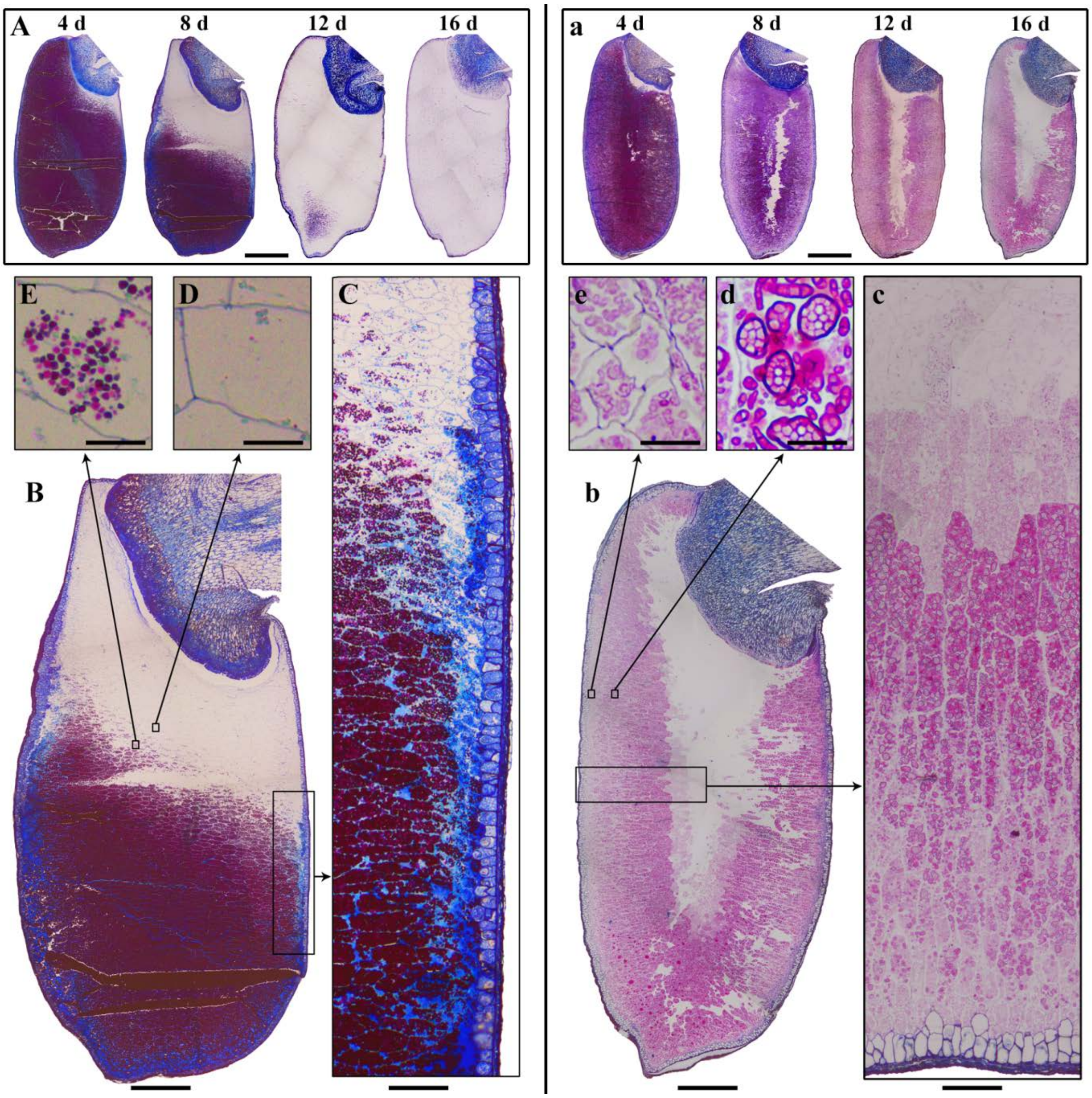

Additional file 1: Figure S7. ***In situ*** **degradation of starch granules in endosperm of rice at different days after imbibition** (cited from Pan et al. [59]). The LR White resin section is prepared using the improved method of whole section of mature kernel, and counterstained with periodic acid-Schiff's and toluidine blue O. (A-E) Wild type rice kernels; (B-E) The magnification of germinated kernels at 8 days after imbibition, showing starch degradation in different regions of endosperm. (a-e) High-amylose rice kernels with inhibition of starch branching enzyme I and IIb; (b-e) The magnification of germinated kernel at 16 days after imbibition, showing the degradation of heterogeneous starch granules in different regions of endosperm. Scale bar = 1 mm (A, a), 500 μm (B, b), 100 μm (C, c), and 20 μm (D, E, d, e).
